# Lithium chloride attenuates root resorption during orthodontic tooth movement in rats

**DOI:** 10.3892/etm.2013.1410

**Published:** 2013-11-15

**Authors:** YU WANG, SHANG GAO, HUAN JIANG, PENG LIN, XINGFU BAO, ZHIMIN ZHANG, MIN HU

**Affiliations:** 1 Department of Orthodontics, School of Stomatology, Jilin University, Changchun, Jilin 130021, P.R. China; 2 Department of Endodontics, School of Stomatology, Jilin University, Changchun, Jilin 130021, P.R. China

**Keywords:** lithium chloride, orthodontic tooth movement, root resorption

## Abstract

Root resorption is a common side effect of orthodontic treatment. In the current study, lithium chloride (LiCl), a Wnt signaling activator, was examined to determine its effect on root resorption. In total, 10 Sprague Dawley rats were randomly allocated into the experimental group (EG) and control group (CG). Each group consisted of five subjects. By using closed nickel-titanium coil springs, a 50-g force was applied between the upper incisors and the maxillary right first molars in order to mimic orthodontic biomechanics in the EG and CG for 14 days. During the 14 days, the EG rats were gavage-fed 200 mg/kg LiCl every 48 h. Next, digital radiographs were captured using a micro-computational tomography scanner. The movement of the maxillary first molars and the root resorption area ratio were measured electronically on the digital radiographs. The outcomes were analyzed using ANOVA. Following 14 days of experimental force application, all rats had spaces of varying sizes between the first and second right maxillary molars. The average distance measured in the CG was slightly higher than in the EG, however, the difference was not found to be statistically significant (P=0.224). Root resorption craters were observed in the groups following the experiment. Rough cementum areas were observed on the mesial surface of the distobuccal and distopalatal roots. The mean root resorption area ratio of CG was significantly greater than EG (P<0.05). Results of the present study indicate that LiCl can attenuate orthodontically induce root resorption during orthodontic tooth movement. The effect of LiCl on tooth movement is insignificant.

## Introduction

Orthodontic tooth movement is based on the response of biological tissue towards a mechanical force. The movement occurs as a result of alveolar bone remodeling through the prolonged application of a controlled force. The applied force induces bone resorption and bone formation on the pressure and tension zones, respectively. In order to move a tooth in the intended direction, bone resorption on the pressure side of the socket wall creates space for the advancing tooth, while bone deposition on the tension side maintains a progressively advancing socket wall behind the moving tooth (1,2). Bone remodeling involves the resorption of bone tissue with the simultaneous formation of new bone tissue; the two processes are in dynamic equilibrium in normal bone (3). During orthodontic tooth movement, alveolar bone resorption at the area of compression occurs through osteoclastic activities by osteoclasts, consequently creating lacunae which are later occupied by osteoblasts to cover the cavity. There are two processes in bone resorption, the solubilization of minerals and the degradation of the organ matrix, largely consisting of type I collagen. These processes are mediated by proteolytic enzymes, including matrix metalloproteinases and lysosomal cysteine proteinases (4). In the tension region, new bone is formed as a result of mechanical force during orthodontic treatment and osteoblasts differentiate from the local precursor cells. Then osteoid is formed by mature osteoblasts and is further mineralized with the secretion of calcium ion (5).

Root resorption is a common feature during orthodontic tooth movement. Histological studies have reported that root resorption occurs in 90% of teeth that have been moved orthodontically (6,7). Root resorption, is an unavoidable pathological outcome of orthodontic tooth movement. It is considered to be the result of an inflammatory reaction; thus, certain researchers call this process ‘orthodontically-induced inflammatory root resorption’ (8).

The fundamental component behind the root resorption process is local inflammation induced by mechanical forces which is essential for tooth movement during orthodontic treatment (9). Studies have confirmed that orthodontically-induced root resorption is a part of the hyaline (sterile necrosis) zone elimination process. The first cells to be involved in the removal of necrotic tissue are macrophage-like cells, which may be activated by signals from sterile necrotic tissue, as a result of orthodontic force application (10,11). Osteoclasts and odontoclasts are implicated in the root resorption process (10). In experimental rats, the extent of root resorption is reported to increase only when force reactivation is performed at the peak count of osteoclasts in the involved region (12,13). Several studies have reported the involvement of multinucleated tartrate-resistant acid phosphatase-positive giant cells without ruffled borders in the removal of hyaline tissue. These cells may be early osteoclasts or odontoclasts (preosteoclasts) that become involved in the elimination of necrotic tissue. The cells differentiate into fully developed osteoclasts or odontoclasts within hours following the introduction of a new mechanical stimulus (14–16).

Under normal health conditions, bone tissue undergoes a continuous process of balanced remodeling, regulated by a number of systemic and local factors (17). Wnt ligands are among the local signaling factors implicated in the process of bone remodeling. Therefore, pharmacological modulation of this pathway may affect bone mass. Activation of the canonical Wnt signaling pathway *ex vitro* and *in vivo* may be achieved with lithium chloride (LiCl) (18–20).

Lithium enhances bone formation and improves bone mass in mice, and may do so via the activation of the canonical Wnt pathway (21). The Wnt coreceptor low-density lipoprotein receptor-related protein 5, interacts with the Wnt ligand and members of the Frizzled co-receptor family at the cell surface, transducing the Wnt signal via the canonical pathway and ultimately leading to the nuclear accumulation of β-catenin (22). β-catenin enhances bone formation, through a canonical Wnt signaling cascade. Previous studies have shown tissue cell-specific osteoblast production failure in mice with β-catenin inactivation (23–25), while mice with high levels of β-catenin in their osteoblasts, undergo excessive bone formation with limited levels of osteoclasts (23,26).

Although cementoblasts share certain phenotypical features with osteoblasts, the canonical Wnt pathway inhibits differentiation and promotes the proliferation of cells responsible for laying down cementum on root surface (27). LiCl enhances bone formation by inhibiting glycogen synthase kinase-3 (GSK-3), an enzyme that phosphorylates β-catenin in the cytoplasm, targeting it for ubiquitination and degradation. The process increases the concentration of β-catenin and augments bone formation through the activation of the canonical Wnt signaling pathway (18–20).

Little is known of the consequences of LiCl on dental tissue (15,16,27), therefore the aim of the current study was to uncover the effect of LiCl on root resorption during orthodontic tooth movement in rats.

## Materials and methods

### Animals

Ethical approval for this study was obtained from the Institutional Animal Welfare Committee of the Chaoyang district and the Jilin University Animal Care Commissioner (Changchun, China). In total, 10 Sprague Dawley rats were purchased from the Animal Laboratory of Jilin University. The study involved healthy 8-week-old male rats, weighing 200±10 g at the start of experiment. The rats were randomly allocated into an experimental group (EG) or control group (CG). There were 5 rats in each group.

**Figure 1:**
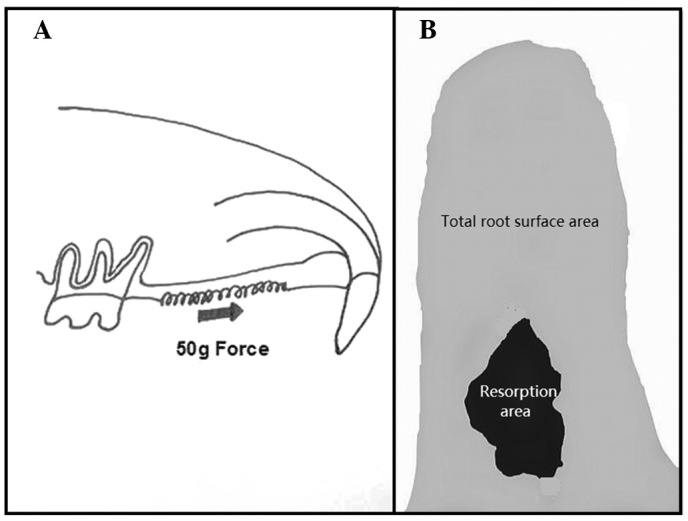
Schematic diagrams for the rat tooth movement model and root resorption. (A) The nickel-titanium coil spring between the first molar and incisors was activated to create a 50-g force. (B) Root resorption area ratio was calculated by dividing the total root surface area (gray) by the resorption area (black).

With the use of closed nickel-titanium (NiTi) coil springs (Shengmate Technology Co. Ltd. Beijing, China), a 50-g force was applied between the upper incisor and the first molars in order to mimic orthodontic biomechanics in the experimental and control groups. The appliances, consisting of a NiTi coil spring tied with stainless steel wires between the maxillary incisors and right first molar, were placed in all subjects under general anesthesia (Fig. 1). A tension gauge (Tianmei New Environmental Material Co. Ltd., Nanchang City, China) was used to measure the force of the springs prior to inserting them into the mouth of the rat. The ends of the ligature wires were covered with composite resin to prevent the appliances from loosening.

All subjects in the EG were gavage-fed 200-mg/kg LiCl every 48 h during the experiment. The CG was not treated medically. The rats were sacrificed after 14 days using an anesthetic overdose.

**Figure 2:**
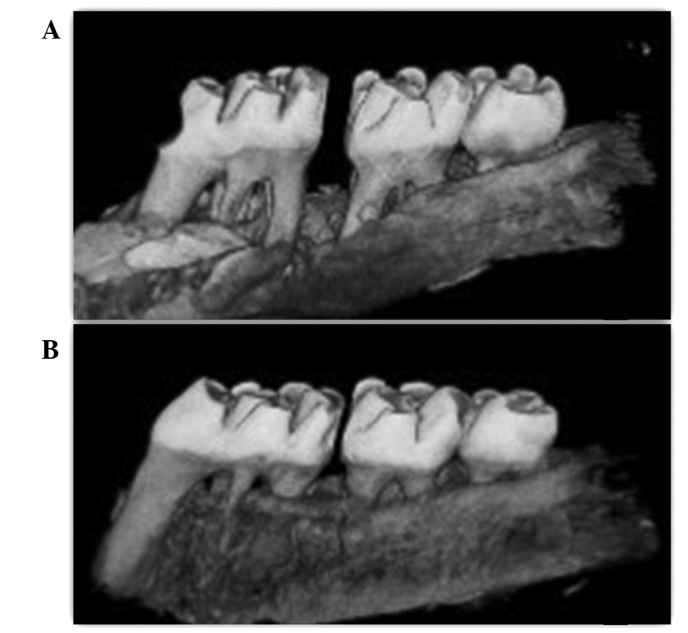
Micro-computed tomography images for tooth movement in the (A) experimental and (B) control groups.

Tooth movement was assessed using ZKKS-MCT-Sharp micro-computed tomography (Zhongke Kaisheng Medical Technology Co. Ltd. Guangzhou, China). The rats were scanned at the same distance and orientation in order to obtain the lateral cephalographs of the maxillary molars with the surrounding alveolar bone (Fig. 2).

### Measurement

**Figure 3:**
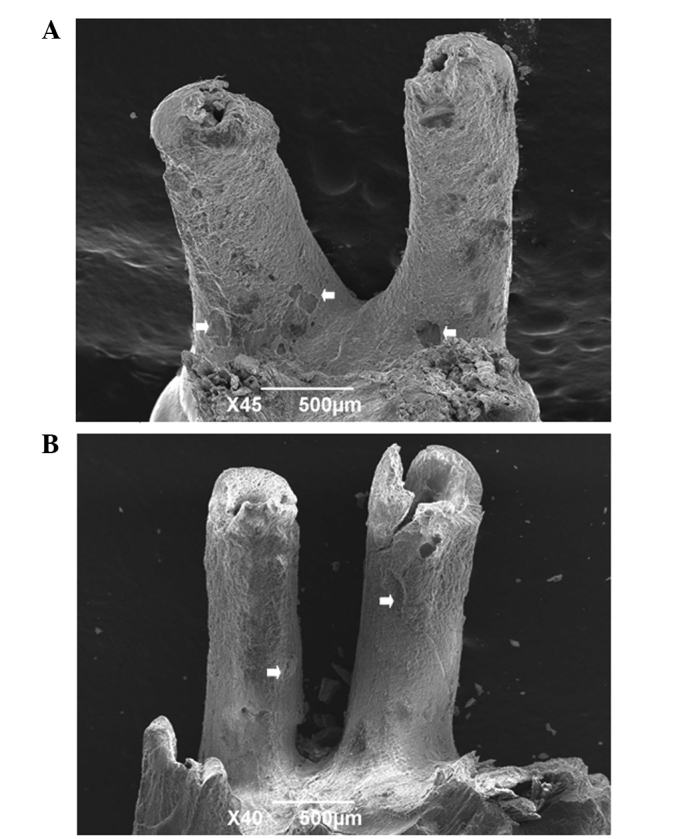
Scanning electron microscope images of tooth surface in the (A) control and (B) experimental groups. The resorption area is indicated by a white arrow.

The distance between the nearest points of the first and second molars was measured on the digital radiographs of each subject using ImageJ software version 1.44; (National Institutes of Health, Bethesda, MD, USA) to determine the amount of tooth movement. After measuring tooth movement, the maxillary right first molar and the surrounding alveolar bone were extracted *in toto* for root resorption assessment. The samples were soaked in 5% sodium hypochlorite solution for 12 h. Subsequently, the alveolar bone was removed to expose the five roots of the first molar. In order to obtain clear root surfaces, the distobuccal and distopalatal roots were carefully cleaned to remove the remnants of periodontal ligaments. The teeth were dried and scanned for resorption area examination. The resorption areas on the digital radiographs of the mesial surfaces of the distal roots were measured using ImageJ software. The resorption area and the entire mesial surface area of the two distal roots were measured separately using the same software (Fig 3).

### Statistical analysis

The resorption area ratio was obtained by dividing the resorption crater area by the total surface area. The same researcher measured all subjects, and every measurement was repeated three times. The mean value was used in the final analysis. One-way analysis of variance was performed using SPSS version 17 (SPSS Inc., Chicago, IL, USA) to compare the groups. P<0.05 was considered to indicate a statistically significant result.

## Results

### Observations

The rats survived until the end of the experiment. Prior to the experiment, none of the rats had a space between the first and second molar crowns. Following 14 days of experimental force application, all subjects had spaces of varying sizes between the first and second right maxillary molars. The space appeared in the third day after force loading and became apparent after 7 days. The average distance measured in the CG was slightly higher than in the EG. However, the difference was not considered statistically significant (P=0.224; Table I).

Roots of the target molars in all subjects were successfully separated from the alveolar bone. Root resorption craters with different forms were observed in the two groups following the experiment, mainly in the cervical and middle one-third of the root. Rough cementum areas were observed on the mesial surface of the distobuccal and distopalatal roots. The mean root resorption area ratio of the CG was significantly greater than that of the EG (P<0.05).

## Discussion

Previous studies have demonstrated the ability of LiCl to enhance bone formation via the canonical Wnt/β-catenin signaling pathway. LiCl has been used for decades for the treatment of bipolar disorder by increasing β-catenin signaling through the inhibition of GSK-3β (18,28). The canonical Wnt/β-catenin pathway increases bone mass in several ways, including the renewal of stem cells, stimulation of preosteoblast replication, induction of osteogenesis and inhibition of osteoblast and osteocyte apoptosis (29).

Since orthodontic tooth movement involves the repeated process of alveolar bone remodeling (30,31), LiCl has the potential to affect tooth movement during orthodontic treatment by affecting the process of bone formation. Osteoclasts, are involved in resorbing the alveolar bone at pressure areas, which appear in the direction of the applied force, while osteoblasts are involved in new bone formation at tension areas, on the opposite side (30,32).

Osteoclasts and odontoclasts are implicated, with other cells, in the orthodontically-induced inflammatory root resorption process (14–16).

LiCl enhances bone formation through the canonical Wnt/β-catenin pathway. Studies have shown that mice with high levels of β-catenin in osteoblasts form excessive bone substances with limited osteoclasts (23,26). Since osteoclasts and odontoclasts share a variety of common features, it is logical that LiCl may reduce root and alveolar bone resorption in the pressure zone. This may be achieved through the inhibition of osteoclasts and odontoclasts, which play key roles in resorption during orthodontic tooth movement. In addition, the enhancement of bone formation on the pressure side may slow down tooth movement, which requires bone resorption to give way, ahead of the moving tooth.

The current study observed a significant reduction in the root resorption area ratio among the EG rats compared with the CG rats (P<0.05), indicating that LiCl is capable of suppressing root resorption during orthodontic treatment. This may be due to the inhibitory effects of LiCl on osteoclastic cells which are implicated in the orthodontically-induced inflammatory root resorption process.

A variety of drugs that have been reported to limit the inflammation process induced by orthodontic biomechanics, tend to suppress root resorption and hinder the movement of teeth by interfering with the resorption process on the pressure side (34–36). The tooth movement measured in the current study was higher in the CG compared with the EG. However, the difference was not statistically significant. To the best of our knowledge, there has been no studies published on the effects of LiCl on root resorption and tooth movement with a comparable study design. However, other studies have investigated the effect of several medicaments on orthodontic tooth movement (38,39).

The topical application of bisphosphonates, which are potent bone resorption inhibitors capable of inducing osteoclast apoptosis (40), has been reported to suppress orthodontic tooth movement (41,42). Non-steroidal anti-inflammatory drugs (NSAIDs) have been reported to suppress root resorption. NSAIDs tend to limit tooth movement through the inhibition of the periodontal inflammatory response induced by orthodontic force (34). Histological changes were not assessed in the current study. Histological analysis is likely to have provided valuable information regarding the effect of LiCl on periodontal tissue during orthodontic tooth movement and is acknowledged to be one of the shortcomings of this study. However, the radiological findings of the study may be used as a foundation for further investigations on the potential benefits of LiCl in contemporary orthodontic clinical practice.

In conclusion, LiCl attenuates orthodontically-induced root resorption during orthodontic tooth movement. The effect of LiCl on tooth movement is insignificant.

## Figures and Tables

**Table I:** Tooth movement and root resorption following 14 days of treatment.

Variable	Experimental group	Control group	P-value
Tooth movement, mm	0.1120±0.061	0.1755±0.072	0.224
Root resorption area ratio	0.0491±0.027	0.1535±0.106	0.046*

* P<0.05.
